# AI-augmented reconstruction provides improved image quality and enables shorter breath-holds in contrast-enhanced liver MRI

**DOI:** 10.1186/s41747-025-00582-1

**Published:** 2025-05-01

**Authors:** Francesca Castagnoli, Mihaela Rata, Joshua Shur, Georgina Hopkinson, Alison Macdonald, David Stockton, Marcel Dominik Nickel, Stephan Kannengiesser, Christina Messiou, Dow-Mu Koh, Jessica Mary Winfield

**Affiliations:** 1 Department of Radiology, Royal Marsden Hospital, London, UK; 2 Division of Radiotherapy and Imaging, The Institute of Cancer Research, London, UK; 3 Department of Physics, Royal Marsden Hospital, Sutton, UK; 4 MR Application Predevelopment, Siemens Healthineers AG, Erlangen, Germany

**Keywords:** Artifacts, Artificial intelligence, Liver neoplasms, Magnetic resonance imaging, Neural networks (computer)

## Abstract

**Background:**

To compare liver image quality and lesion detection using an AI-augmented T1-weighted sequence on hepatobiliary-phase gadoxetate-enhanced magnetic resonance imaging (MRI).

**Methods:**

Fifty patients undergoing gadoxetate-enhanced MRI were recruited. Two T1-weighted Dixon sequences were utilized: a 17-s breath-hold acquisition and an accelerated 12-s breath-hold acquisition (reduced phase resolution), both reconstructed using neural network (NN) and iterative denoising (ID), NN-alone, ID-alone, and the standard method. Contrast-to-noise ratio (CNR) was assessed quantitatively for all series (ANOVA). Two blinded radiologists independently analyzed three image sets: 17-s acquisition reconstructed with NN and ID (17-s NN + ID), 12-s acquisition reconstructed with NN and ID (12-s NN + ID), and 17-s acquisition with standard reconstruction (17-s standard). Overall image quality, qualitative CNR, lesion edge sharpness, vessel edge sharpness, and respiratory motion artifacts were scored (4-point Likert scale) and compared (Friedman test). Lesion detection was compared between 12-s NN + ID and 17-s standard reconstructions (Wilcoxon signed-rank test).

**Results:**

Quantitative liver-to-portal vein CNR was significantly higher for 17-s NN + ID than 17-s standard or 17-s NN-alone images (*p* = 0.001). Scores for overall image quality, qualitative CNR, vessel edge sharpness, and lesion edge sharpness were significantly higher for 17-s NN + ID and 12-s NN + ID than standard reconstruction (*p* < 0.001); there was no significant difference between 17-s and 12-s NN + ID. There was no significant difference in respiratory motion artifacts and number of lesions or diameter of the smallest detected lesion using 12-s NN + ID or 17-s standard reconstruction.

**Conclusion:**

AI-augmented reconstructions can improve image quality while reducing breath-hold duration in T1-weighted hepatobiliary-phase gadoxetate-enhanced MRI, without compromising lesion detection.

**Relevance statement:**

AI-augmented reconstruction of T1-weighted MRI improves image quality and lesion detection in hepatobiliary phase liver imaging, reducing breath-hold duration without compromising clinical lesion detection.

**Key Points:**

- Liver-to-portal vein CNR was significantly higher for 17-s NN + ID.
- AI-augmented reconstructions scored higher for image quality, contrast-to-noise, vessel-edge, and lesion-edge sharpness.
- No significant difference in lesion detection between 12-s NN + ID and 17-s standard reconstructions.

**Graphical Abstract:**

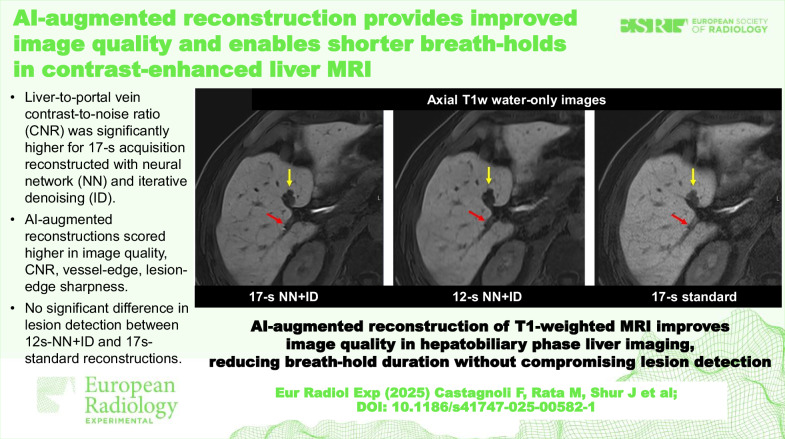

## Background

Magnetic resonance imaging (MRI) of the liver can provide multiparametric information without radiation exposure [1]. T1-weighted imaging, which typically uses gradient-echo techniques acquired during a breath-hold, is one of the key sequences in liver MRI and is used during precontrast, arterial, venous, and delayed postcontrast phases to detect and characterize focal liver lesions [2, 3]. However, it is limited by some technical challenges, such as the time required for each breath-hold acquisition, which can lead to motion artifacts [4].

To reduce the MRI scan time for each breath-hold acquisition, many studies have reported the advantages of acceleration techniques such as parallel imaging, partial Fourier reconstruction, and reduced phase-encode resolution [5, 6]. However, these can lead to blurring and reduction of signal-to-noise ratio in images, particularly at high acceleration factors, which may impair interpretation, particularly for detecting small lesions. Although standard acceleration techniques are commonly used, the need for breath holding (typically ~ 17 s) remains challenging for certain patients. Free-breathing radial T1-weighted abdominal imaging sequences could be an option; however, studies have indicated that, when available, breath-held Cartesian T1-weighted gradient-echo imaging is preferred over free-breathing T1-weighted in abdominal MRI [7, 8].

Recent innovations have led to the use of artificial intelligence (AI) and deep learning in abdominal MRI [9–12]. AI-based reconstruction techniques have been proposed for diffusion-weighted imaging using parallel imaging with sensitivity encoding, respiratory-triggered fast spin-echo T2-weighted, breath-hold fast advanced spin-echo T2-weighted, and three-dimensional GRE T1-weighted sequences. These methods have been demonstrated to improve the detection rate of clinically relevant liver lesions by enhancing image quality and resolution, while simultaneously reducing acquisition times in hepatocellular carcinomas and other primary liver cancers [13–16]. Recently, a new T1-weighted sequence, derived from applying a neural network (NN) trained on high-resolution images to conduct interpolation and partial Fourier reconstruction, in addition to iterative denoising (ID), has been introduced to reduce liver scan times while maintaining image quality [17, 18].

Hence, the aim of this study was to compare image quality and lesion detection using such AI-augmented hepatobiliary phase T1-weighted MRI sequence compared to a standard reconstruction.

## Methods

### Study population

**Fig. 1:**
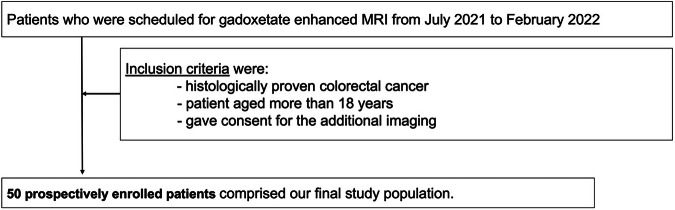
Diagram showing the number of patients and the inclusion criteria of our study

This prospective study was approved by our institutional review board and national research ethics committee; verbal consent was obtained from all patients for additional imaging as part of routine clinical examinations. As a power calculation, a sample size of 16 subjects allows detection of an effect size of 0.8 in image quality score with 90% power and two-sided α = 0.1 [19]. However, to enhance our experience with the sequence and to obtain a more comprehensive range of examples, 50 consecutive patients undergoing gadoxetate-enhanced MRI on one MR scanner between July 2021 and February 2022 were enrolled. The patient inclusion criteria were as follows: (1) histologically proven colorectal cancer; (2) age ≥ 18 years; (3) given consent for additional imaging (Fig. 1). Histology was obtained either through biopsy or from surgical specimens. Patient demographics of the final included cohort are summarized in Table 1.

**Table 1:** Patients’ demographics

Characteristics (*n* = 50)	
Age (years)	57 (36–81)
Sex (female/male)	18/32
Height (cm)	173 (130–194)
Body weight (kg)	75 (50–138)
Body mass index (kg/m^2^)	24.8 (16.7–43.2)

Median values are presented with minimum and maximum values in parentheses

### MRI protocol

Patients were imaged on a 1.5-T scanner (MAGNETOM Aera, Siemens Healthineers, Erlangen, Germany). Patients underwent routine clinical liver MRI, and research sequences were performed at the end of the examination in the hepatobiliary phase, 20 min after contrast administration of gadoxetate (Gd-EOB-DTPA, Primovist^TM^, Bayer, Leverkusen, Germany, 0.25 mmol/mL, administered at 0.1 mL/kg). The study sequences consisted of two T1-weighted gradient-echo Dixon sequences. One sequence (17-s breath-hold) was selected to match the acquisition parameters of the routine protocol at our institution, which includes acceleration using ‘Controlled Aliasing in Parallel Imaging Results in Higher Acceleration’−CAIPIRINHA, reduced phase resolution, reduced slice resolution, and slice partial Fourier reconstruction. The other study sequence was further accelerated by reducing the phase resolution from 75% to 50% to obtain a 12-s breath-hold acquisition. Full parameters of both study sequences are shown in Table 2. Half of the patients had the 17-s followed by the 12-s acquisition, and half had the 12-s followed by the 17-s acquisition to ensure that the breath-hold pattern of the patient had a similar impact on both.

**Table 2:** Imaging sequence parameters

	17-s breath-hold acquisition	12-s breath-hold acquisition
Sequence	Three-dimensional fast low-angle shot with Dixon reconstruction	
Slab/slice orientation	Axial	
FOV (read × phase) (mm × mm)	380 × 309	
Slices per slab	72	
Slice thickness (mm)	3.0	
In-plane phase-encoding direction	Anterior–posterior	
Acquired matrix (read)	320	
Phase resolution (%)	75	50
Reconstructed pixel size (read × phase) mm × mm	0.6 × 0.6	
Echo times (ms)	2.39, 4.77	
Repetition time (ms)	6.68	
Flip angle (degrees)	30	
Parallel imaging	CAIPIRINHA, 2 × 2 re-ordering shift = 1	
Slice partial Fourier	6/8	
Slice resolution (%)	50	
Breathing instructions	Breath hold on exhalation	
Acquisition time (s)	17	12

The 17-s breath-hold acquisition replicated the parameters of the routine clinical protocol at our institution. The 12-s breath-hold acquisition was accelerated by reducing the phase resolution from 75% to 50% in order to reduce the breath-hold duration

*CAIPIRINHA* Controlled aliasing in parallel imaging results in higher acceleration, *FOV* Field of view, *PE* Phase encoding

**Fig. 2:**
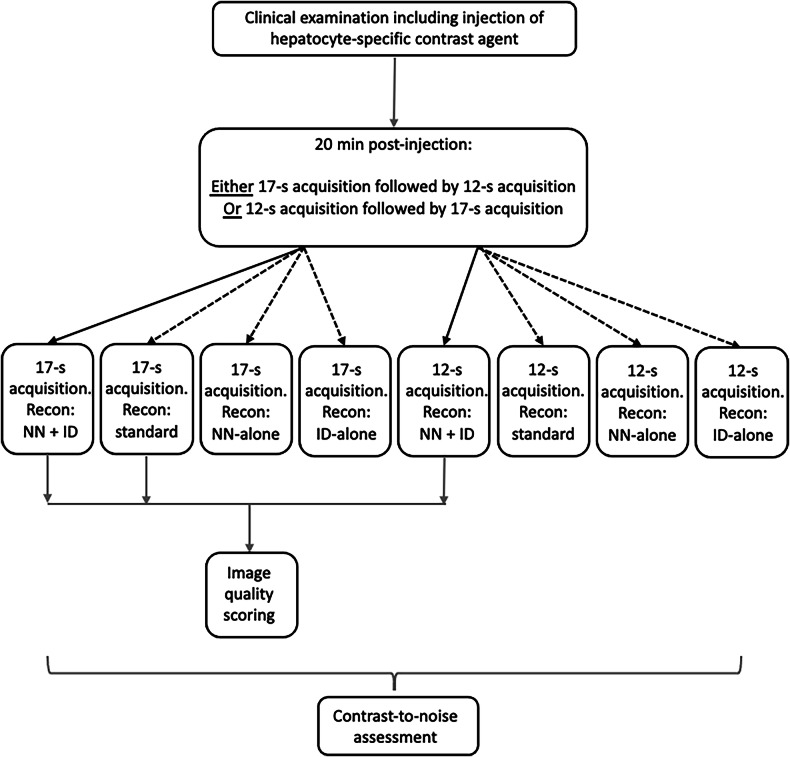
Study workflow. ID, Iterative denoising; NN, Neural networks

Data were reconstructed inline using a research reconstruction algorithm provided by Siemens Healthineers, consisting of a NN to perform interpolation and partial Fourier reconstruction, combined with ID for both sequences (continuous arrows in Fig. 2). The NN described by Almansour et al [17] was trained on high-resolution images and uses a sequence of convolutions and leaky rectified linear units with three skip connections in order to conduct interpolation in the two phase-encoding directions of the three-dimensional gradient-echo sequence. The ID method described by Kannengiesser et al [18] operates on the complex-valued image data and employs an iterative process of wavelet transformation followed by thresholding and weighted recombination to achieve spatially-adapted denoising depending on the local noise level in the images. At the end of the examination, additional reconstructions of the same raw data (dotted arrows in Fig. 2) were performed on the scanner using three options for each sequence: NN-alone, ID-alone and standard reconstruction (zero-filling interpolation without NN or ID); the standard reconstruction replicated the conventional reconstruction used in the manufacturer’s clinically provided imaging. Thus, each acquisition (of 17-s or 12-s) generated four reconstructed series. Although the Dixon sequence outputs four types of images, only the ‘water-only’ images from Dixon reconstructions were used for further analysis, as these are typically used for image interpretation in clinical practice. The 12-s standard reconstruction was also acquired but exhibited very poor image quality, rendering it unsuitable for meaningful evaluation; therefore, it was not included in further analyses.

### Quantitative CNR analysis

Contrast-to-noise ratio (CNR) was assessed quantitatively in all eight series. A 1-cm diameter circular region-of-interest was placed within the largest lesion, in the adjacent normal liver, and in the portal vein to assess the visualization of all liver structures in the hepatobiliary phase by a senior MR radiographer (D.S.), with the supervision of an abdominal radiologist (J.S.). The mean and standard deviation of the signal were measured for each region-of-interest (Osirix MD, v9.0, Pixmeo SARL, Bernex, Switzerland). CNR between the liver and portal vein was quantified as the difference in mean signals divided by the standard deviation of the signal in the liver. CNR between liver and lesion (difference in mean signals divided by standard deviation of signal in the liver) was assessed in patients who had at least one lesion larger than 1 cm.

### Image quality analysis

The water-only series generated by the 17-s acquisition reconstructed with NN and ID (17-s NN + ID), the 12-s acquisition reconstructed with NN and ID (12-s NN + ID) and the 17-s acquisition with standard reconstruction (17-s standard reconstruction) were analyzed independently by two board-certified radiologists (J.S. and F.C., with 10 years and 6 years of experience of liver MRI reporting, respectively), who were blinded to individual subject characteristics and image acquisition and reconstruction methods, with image series presented in a random order. All readings were performed on a ‘Picture archiving and communication system’−PACS workstation (IDS7, version 20, Sectra, Linköping, Sweden).

Overall image quality, qualitative CNR, lesion edge sharpness, vessel edge sharpness, and respiratory motion artifacts were scored on a 4-point Likert scale (1 = unacceptable/non-diagnostic; 2 = adequate; 3 = good; 4 = excellent).

### Lesion detection

Clinical lesion detection was assessed by identifying and counting all focal liver lesions and measuring the diameter of the smallest lesion on the 12-s NN + ID acquisition and 17-s standard reconstruction. The detected lesions comprised both liver metastases from colorectal cancer and benign lesions, including cysts and hemangiomas. The two readers (J.S. and F.C.) were blinded to acquisition and reconstruction; the series was randomized with a four-week washout period between the reading sessions to reduce recall bias. Five patients were excluded from this analysis as full liver coverage was not achieved in one of the series due to variation in breath holding between the two acquisitions.

### Statistical analysis

Differences in quantitative CNR between series were assessed using ANOVA. The Friedman test was used to compare image quality scores, and post-hoc analysis (pairwise Wilcoxon signed-rank tests with Bonferroni correction) was used to identify significantly different imaging series. Following Kolmogorov–Smirnov tests for normality, Wilcoxon signed-rank tests were used to compare a number of lesions recorded and the diameter of the smallest lesion for each reader. Statistically significant differences were defined as *p* < 0.05 (and as 0.05 divided by a number of comparisons where Bonferroni corrections were applied).

The inter-rater percentage agreement was used to evaluate the image quality scores from the two readers. The distinction between an excellent and good examination is probably less clinically important than the difference between a good and adequate examination. As a result, the qualitative scores were grouped into two categories: excellent/good and adequate/non-diagnostic, and analysed as percentage agreement.

All statistical analyses were performed with MATLAB (2021, The MathWorks, Inc., Natick, MA, USA).

## Results

### Patient demographics

Fifty patients who underwent clinical gadoxetate MRI were included, 32 males and 18 females, with a median age of 57 years (range 36–81 years) (Table 1).

### Quantitative CNR analysis

**Fig. 3:**
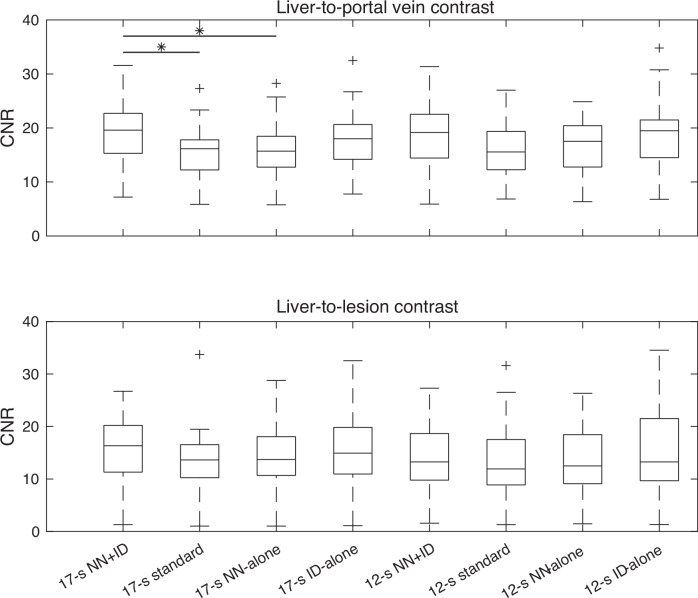
Boxplots showing quantitative contrast measurements between the liver and the portal vein (top row), and the liver and the lesion (bottom row). Significant differences (*p* ≤ 0.001) are denoted by an asterisk (*); other differences were not significant. ID, Iterative denoising; NN, Neural networks

Twenty-four patients had lesions larger than 1 cm. The liver-to-portal vein CNR was significantly higher on the 17-s NN + ID images compared with those obtained using the 17-s standard reconstruction or 17-s NN-alone (*p* = 0.001) (Fig. 3). All other comparisons were not significantly different (Fig. 3). Median CNR values are shown in Table 3.

**Table 3:** Quantitative CNR between liver and portal vein (top row) and liver and lesion (bottom row)

	17-s NN + ID	17-s standard	17-s NN-alone	17-s ID-alone	12-s NN + ID	12-s standard	12-s NN-alone	12-s ID-alone
Liver-to-portal vein (*n* = 50 patients)	19.6 (15.3–22.7)	16.1 (12.2–17.8)	15.7 (12.7–18.5)	18.0 (14.2–20.7)	19.2 (14.4–22.5)	15.6 (12.3–19.3)	17.5 (12.8–20.4)	19.5 (14.5–21.5)
Liver-to-lesion (*n* = 24 patients)	16.3 (11.3–20.2)	13.6 (10.2–16.5)	13.7 (10.7–18.1)	14.9 (10.9–19.8)	13.3 (9.8–18.7)	11.9 (8.9–17.5)	12.5 (9.1–18.5)	13.2 (9.7–21.5)

Results show median CNR (interquartile range). Data as shown in Fig. 3

*ID* Iterative denoising, *NN* Neural networks

### Image quality analysis

**Fig. 4:**
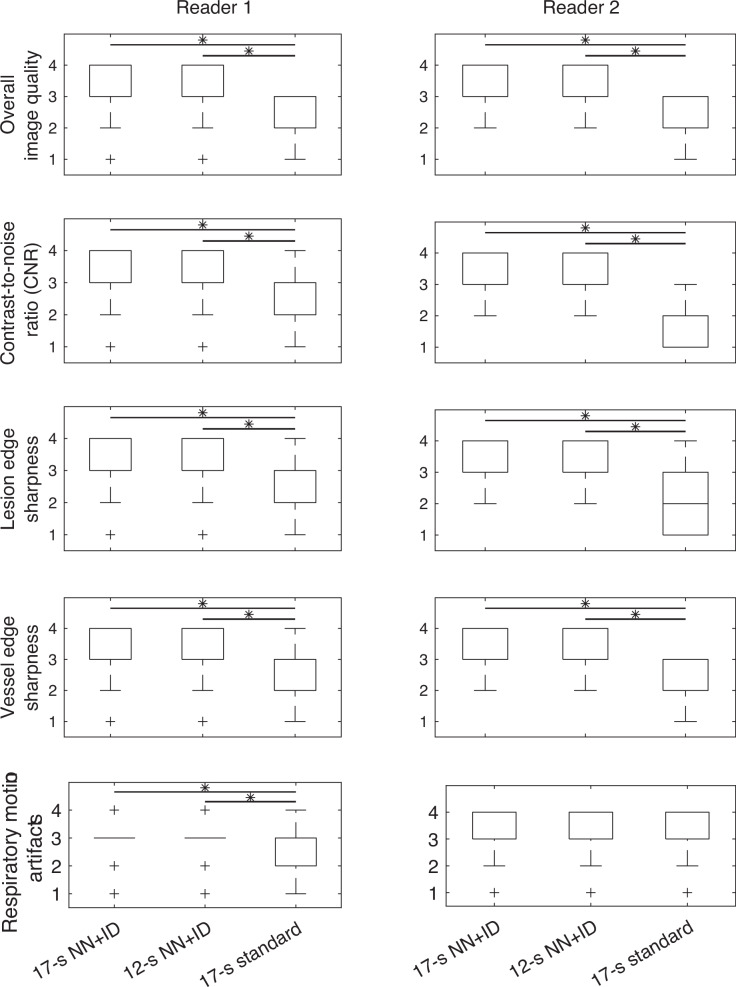
Boxplots showing scores for image quality, CNRs, vessel edge sharpness, lesion edge sharpness, and respiratory motion artifacts for the 17-s and 12-s acquisitions with NN + ID and the 17-s acquisition with standard reconstruction. Significant differences (*p* ≤ 0.001) are denoted by an asterisk (*); other differences were not significant. ID, Iterative denoising; NN, Neural networks

**Fig. 5:**
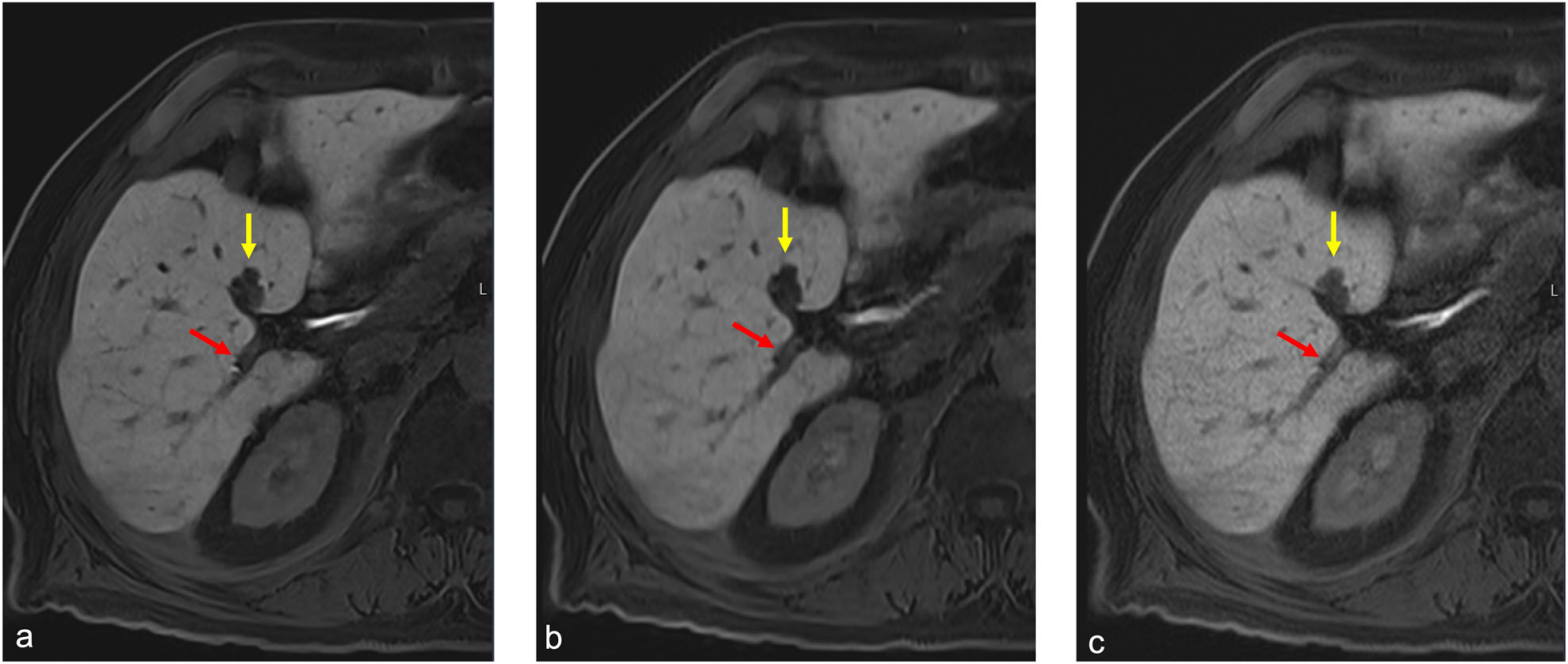
Axial T1-weighted water-only reconstructions from Dixon imaging in the hepatobiliary phase following administration of gadoxetate. Scores were higher for image quality, CNR, vessel edge sharpness (red arrow), and lesion edge sharpness (yellow arrow) for the 17-s (**a**) and 12-s (**b**) acquisitions using NN + ID compared with the 17-s acquisition with standard reconstruction (**c**)

There were significantly higher median scores for overall image quality, CNR, vessel edge sharpness and lesion edge sharpness for the 17-s and 12-s acquisitions using NN + ID compared with 17-s standard reconstruction (*p* < 0.001), but with no significant difference between 17-s and 12-s acquisitions using NN + ID (Fig. 4; example shown in Fig. 5; Supplementary Table S1) for both readers. There were significantly higher scores for respiratory motion artifacts (*i.e*., images less affected by respiratory motion artifacts) for the 17-s and 12-s acquisitions using NN + ID compared with 17-s standard reconstruction for Reader 1 (*p* = 0.001 for both) but not for Reader 2. After binarizing the scores into excellent/good and adequate/non-diagnostic categories, the inter-rater percentage agreement for all scores ranged from 58% to 92%.

Exploratory subgroup analysis comparing patients who had the 17-s followed by the 12-s acquisition, *versus* patients who had the 12-s followed by the 17-s acquisition, showed no difference in any image quality scores (overall image quality, qualitative CNR, lesion edge sharpness, vessel edge sharpness, respiratory motion artifacts) between the two groups for either reader (*p* = 0.096–1.00).

### Lesion detection

**Fig. 6:**
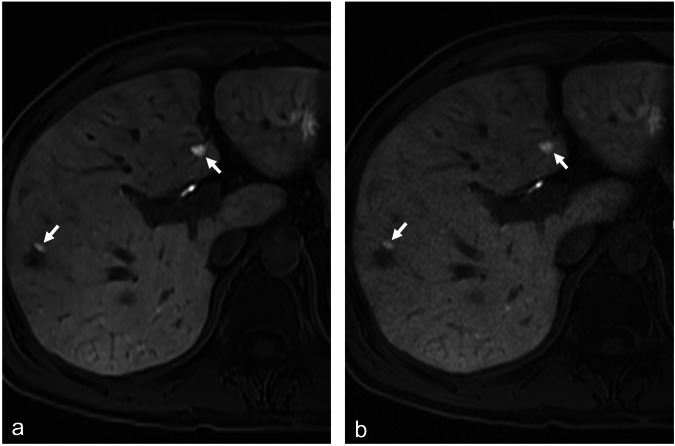
Sixty-four-year-old patient with colorectal cancer. **a** T1-weighted 12-s acquisition NN + ID; **b** T1-weighted 17-s acquisition, standard reconstruction. In both acquisitions, multiple small T1-hyperintense regenerative focal nodular hyperplasia (FNH)-like nodules following oxaliplatin chemotherapy are apparent (white arrows). Even though the 12-s NN + ID image has better image quality, the nodules are visible in both series. ID, Iterative denoising; NN, Neural networks

The total number of lesions recorded is shown in Supplementary Table S2 for each reader, for each imaging series. For clinical lesion detection, 45 out of 50 patients were included in the analysis of lesion detection, with the smallest lesion measuring 1 mm in diameter. Median and IQR numbers of lesions detected by each reader for each series are reported in Supplementary Table S2. There was no significant difference between the number of lesions and the diameter of the smallest lesion identified using 12-s NN + ID compared with 17-s acquisition standard reconstruction for either reader (*p* ≥ 0.0.05/4) (example shown in Fig. 6).

## Discussion

In this liver MRI study, both the 17-s and 12-s acquisitions using NN + ID achieved significantly higher scores for image quality, CNR, vessel edge sharpness, and lesion edge sharpness compared to standard reconstruction, with no significant differences observed between the 17-s and 12-s NN + ID acquisitions. Even though the number of lesions and diameter of smallest lesion identified using the 12-s NN + ID acquisition and 17-s acquisition with standard reconstruction were comparable, the excellent image quality attained using the 12-s acquisition suggests that NN + ID can enable reduced data acquisition, thus decreasing breath-hold duration and making examinations more tolerable for patients, whilst providing improved image quality.

The use of NN + ID improved image quality with increased CNR and edge sharpness for all readers. However, there were discrepant results for respiratory motion artifacts between the 17-s and 12-s acquisitions using NN + ID and standard reconstruction between the two readers, with statistically significant results for Reader 1 but not for Reader 2. This may be explained by the difference in experience between the two readers and by a degree of subjectivity as to what constitutes a motion artifact, which was not explicitly defined.

Several studies have shown the importance of hepatocyte-specific contrast-enhanced T1-weighted liver MRI. In fact, this approach provides information crucial for treatment decision-making, post-therapy follow-up, and functional assessment of the liver and the biliary system for both primary and secondary liver malignancies [20–22]. Nonetheless, despite recent advances in imaging techniques aimed to improve image quality [23, 24], obtaining high-quality T1-weighted imaging is not always feasible in clinical practice, as patients with liver cirrhosis or colorectal liver metastases are often elderly and with limited breath-holding ability [25].

Significantly improved results in terms of quantitative and qualitative analysis for T1-weighted images are in line with previous studies [24, 26]. In particular, Wessling et al [27] reported a significant improvement of image quality, noise, and sharpness while reducing acquisition time in abdominopelvic MRI with the use of a deep learning-based super-resolution algorithm including a partial Fourier technique. Similar results have been found by Chaika et al [28], who investigated the potential role of a deep learning-based super-resolution reconstruction with ID in pancreatic MRI. They reported positive impact on image quality, noise and artifacts level, contrast of organ, as well as conspicuity of vessels and pancreatic ducts.

According to the retrospective study of high-risk hepatocellular carcinoma patients by Kim et al [29], gadoxetate-enhanced liver MRI images with AI-augmented reconstruction significantly improved overall image quality, lesion conspicuity, and liver edge sharpness compared to conventional methods. AI-augmented reconstructed images demonstrated higher sensitivity and detection rates for viable post-treatment hepatocellular carcinoma. Considering the state of the art, we have additionally shown that by implementing an AI-augmented hepatobiliary-phase T1-weighted sequence, it is possible to reduce the duration of breath-holds whilst achieving good image quality, which could increase patients’ compliance during MRI exams.

Despite the encouraging results, there are limitations to the study. First, the study was conducted only in patients undergoing colorectal cancer using a 1.5-T scanner from one manufacturer at a single institution, which may limit the generalizability of the findings to other clinical settings with different MRI protocols, equipment, or patient populations. Second, we acquired and analyzed images only in the hepatobiliary phase; further research will be needed to evaluate the unenhanced and early postcontrast images that may be particularly affected by respiratory motion with gadoxetate [30]. Third, we did not conduct further assessments of the MRI findings concerning benign or malignant criteria, as a comprehensive clinical assessment of lesion benignity or malignancy was beyond the scope of this study and not possible since our evaluation was limited to hepatobiliary phase imaging. Therefore, conclusions regarding the impact of these techniques on specificity cannot be drawn. Similarly, it is not possible to draw conclusions about the differences in the numbers of lesions detected by the different readers, as these differences were not statistically significant, and all lesions (both benign and malignant) were counted without further characterization.

Summarizing, we have shown that AI-augmented reconstruction using a trained NN for data interpolation combined with ID provides improved image quality and enables shorter breath-hold acquisitions in T1-weighted hepatobiliary-phase imaging in liver MRI examinations without compromising lesion detection.

## Supplementary information

**Additional file 1: Supplementary Table 1.** Image quality scores for two readers. **Supplementary Table 2.** Number of lesions detected per patient, for 12-s NN+ID compared with 17-s acquisition standard reconstruction for two readers.

## Data Availability

The datasets used in this study are available from the corresponding author upon reasonable request.

## Abbreviations

AI: Artificial intelligence
CNR: Contrast-to-noise ratio
ID: Iterative denoising
NN: Neural network

## References

[1] Colli A, Fraquelli M, Casazza G et al (2006) Accuracy of ultrasonography, spiral CT, magnetic resonance, and alpha-fetoprotein in diagnosing hepatocellular carcinoma: a systematic review. Am J Gastroenterol 101:513–523. 10.1111/j.1572-0241.2006.00467.x 16542288 10.1111/j.1572-0241.2006.00467.x

[2] Rofsky NM, Lee VS, Laub G et al (1999) Abdominal MR imaging with a volumetric interpolated breath-hold examination. Radiology 212:876–884. 10.1148/radiology.212.3.r99se34876 10478260 10.1148/radiology.212.3.r99se34876

[3] Yu MH, Lee JM, Yoon JH, Kiefer B, Han JK, Choi BI (2013) Clinical application of controlled aliasing in parallel imaging results in a higher acceleration (CAIPIRINHA)-volumetric interpolated breathhold (VIBE) sequence for gadoxetic acid-enhanced liver MR imaging. J Magn Reson Imaging 38:1020–1026. 10.1002/jmri.24088 23559147 10.1002/jmri.24088

[4] Schreiber-Zinaman J, Rosenkrantz AB (2019) Frequency and reasons for extra sequences in clinical abdominal MRI examinations. Abdom Radiol (NY) 42:306–311. 10.1007/s00261-016-0877-6 10.1007/s00261-016-0877-6 27549101

[5] Wang Y (2000) Description of parallel imaging in MRI using multiple coils. Magn Reson Med 44:495–499. 10.1002/1522-2594(200009)44:3<495::aid-mrm23>3.0.co;2-s 10975905

[6] Hamilton J, Franson D, Seiberlich N (2017) Recent advances in parallel imaging for MRI. Prog Nucl Magn Reson Spectrosc 101:71–95. 10.1016/j.pnmrs.2017.04.002 28844222 10.1016/j.pnmrs.2017.04.002 PMC5927614

[7] Hedderich DM, Weiss K, Spiro JE et al (2018) Clinical evaluation of free-breathing contrast-enhanced -T1w MRI of the liver using pseudo golden angle radial k-space sampling. Rofo 190:601–609. 10.1055/s-0044-101263 29534252 10.1055/s-0044-101263

[8] Chandarana H, Block TK, Rosenkrantz AB et al (2011) Free-breathing radial 3D fat-suppressed T1- weighted gradient echo sequence: a viable alternative for contrast-enhanced liver imaging in patients unable to suspend respiration. Invest Radiol 46:648–653. 10.1097/RLI.0b013e31821eea45 21577119 10.1097/RLI.0b013e31821eea45

[9] Herrmann J, Gassenmaier S, Nickel D et al (2021) Diagnostic confidence and feasibility of a deep learning accelerated HASTE sequence of the abdomen in a single breath-hold. Invest Radiol 56:313–319. 10.1097/rli.0000000000000743 33208596 10.1097/RLI.0000000000000743

[10] Ebner M, Patel PA, Atkinson D et al (2019) Super-resolution for upper abdominal MRI: acquisition and post-processing protocol optimization using brain MRI control data and expert reader validation. Magn Reson Med 82:1905–1919. 10.1002/mrm.27852 31264270 10.1002/mrm.27852 PMC6742507

[11] Gassenmaier S, Afat S, Nickel D et al (2021) Application of a novel iterative denoising and image enhancement technique in T1-weighted precontrast and postcontrast gradient echo imaging of the abdomen: improvement of image quality and diagnostic confidence. Invest Radiol 56:328–334. 10.1097/rli.0000000000000746 33214390 10.1097/RLI.0000000000000746

[12] Gassenmaier S, Küstner T, Nickel D et al (2021) Deep learning applications in magnetic resonance imaging: has the future become present? Diagnostics (Basel). 10.3390/diagnostics11122181 10.3390/diagnostics11122181 PMC8700442 34943418

[13] Duan T, Zhang Z, Chen Y et al (2024) Deep learning-based compressed SENSE improved diffusion- weighted image quality and liver cancer detection: a prospective study. Magn Reson Imaging 111:74–83. 10.1016/j.mri.2024.04.010 38604347 10.1016/j.mri.2024.04.010

[14] Kim JH, Yoon JH, Kim SW, Park J, Bae SH, Lee JM (2024) Application of a deep learning algorithm for three-dimensional T1-weighted gradient-echo imaging of gadoxetic acid-enhanced MRI in patients at a high risk of hepatocellular carcinoma. Abdom Radiol (NY) 49:738–747. 10.1007/s00261-023-04124-4 10.1007/s00261-023-04124-4 38095685

[15] Tajima T, Akai H, Yasaka K et al (2022) Clinical feasibility of an abdominal thin-slice breath-hold single-shot fast spin echo sequence processed using a deep learning-based noise-reduction approach. Magn Reson Imaging 90:76–83. 10.1016/j.mri.2022.04.005 35504409 10.1016/j.mri.2022.04.005

[16] Nakaura T, Kobayashi N, Yoshida N et al (2023) Update on the use of artificial intelligence in hepatobiliary MR imaging. Magn Reson Med Sci 22:147–156. 10.2463/mrms.rev.2022-0102 36697024 10.2463/mrms.rev.2022-0102 PMC10086394

[17] Almansour H, Gassenmaier S, Nickel D et al (2021) Deep learning-based superresolution reconstruction for upper abdominal magnetic resonance imaging: an analysis of image quality, diagnostic confidence, and lesion conspicuity. Invest Radiol 56:509–516. 10.1097/rli.0000000000000769 33625063 10.1097/RLI.0000000000000769

[18] Kannengiesser SAR, Mailhe B, Nadar M, Huber S, Kiefer B (2016) Universal iterative denoising of complex-valued volumetric MR image data using supplementary information. ISMRM, p 1779

[19] Faul F, Erdfelder E, Lang AG, Buchner A (2007) G*Power 3: a flexible statistical power analysis program for the social, behavioral, and biomedical sciences. Behav Res Methods 39:175–191. 10.3758/bf03193146 17695343 10.3758/bf03193146

[20] Koh DM, Ba-Ssalamah A, Brancatelli G et al (2021) Consensus report from the 9(th) International forum for liver magnetic resonance imaging: applications of gadoxetic acid-enhanced imaging. Eur Radiol 31:5615–5628. 10.1007/s00330-020-07637-4 33523304 10.1007/s00330-020-07637-4 PMC8270799

[21] Sun HY, Lee JM, Shin CI et al (2010) Gadoxetic acid-enhanced magnetic resonance imaging for differentiating small hepatocellular carcinomas (< or =2 cm in diameter) from arterial enhancing pseudolesions: special emphasis on hepatobiliary phase imaging. Invest Radiol 45:96–103. 10.1097/RLI.0b013e3181c5faf7 20057319 10.1097/RLI.0b013e3181c5faf7

[22] Knowles B, Welsh FK, Chandrakumaran K, John TG, Rees M (2012) Detailed liver-specific imaging prior to pre-operative chemotherapy for colorectal liver metastases reduces intra-hepatic recurrence and the need for a repeat hepatectomy. HPB (Oxford) 14:298–309. 10.1111/j.1477-2574.2012.00447.x 22487067 10.1111/j.1477-2574.2012.00447.x PMC3384849

[23] Yang AC, Kretzler M, Sudarski S, Gulani V, Seiberlich N (2016) Sparse reconstruction techniques in magnetic resonance imaging: methods, applications, and challenges to clinical adoption. Invest Radiol 51:349–364. 10.1097/rli.0000000000000274 27003227 10.1097/RLI.0000000000000274 PMC4948115

[24] Ogasawara G, Inoue Y, Matsunaga K, Fujii K, Hata H, Takato Y (2017) Image non-uniformity correction for 3-T Gd-EOB-DTPA-enhanced MR imaging of the liver. Magn Reson Med Sci 16:115–122. 10.2463/mrms.mp.2016-0012 27385553 10.2463/mrms.mp.2016-0012 PMC5600070

[25] Young Park J, Min Lee S, Sub Lee J, Chang W, Hee Yoon J (2022) Free-breathing dynamic T1WI using compressed sensing-golden angle radial sparse parallel imaging for liver MRI in patients with limited breath-holding capability. Eur J Radiol 152:110342. 10.1016/j.ejrad.2022.110342 35597070 10.1016/j.ejrad.2022.110342

[26] Maennlin S, Wessling D, Herrmann J et al (2023) Application of deep learning-based super-resolution to T1-weighted postcontrast gradient echo imaging of the chest. Radiol Med 128:184–190. 10.1007/s11547-022-01587-1 36609662 10.1007/s11547-022-01587-1 PMC9938811

[27] Wessling D, Herrmann J, Afat S et al (2022) Application of a deep learning algorithm for combined super-resolution and partial fourier reconstruction including time reduction in T1-weighted precontrast and postcontrast gradient echo imaging of abdominopelvic MR imaging. Diagnostics (Basel) 12:2370. 10.3390/diagnostics12102370 36292057 10.3390/diagnostics12102370 PMC9600324

[28] Chaika M, Afat S, Wessling D et al (2023) Deep learning-based super-resolution gradient echo imaging of the pancreas: improvement of image quality and reduction of acquisition time. Diagn Interv Imaging 104:53–59. 10.1016/j.diii.2022.06.006 35843839 10.1016/j.diii.2022.06.006

[29] Park YS, Lee CH, Kim JW, Lee YS, Paek M, Kim KA (2017) Application of high-speed T1 sequences for high-quality hepatic arterial phase magnetic resonance imaging: intraindividual comparison of single and multiple arterial phases. Invest Radiol 52:605–611. 10.1097/rli.0000000000000378 28441159 10.1097/RLI.0000000000000378

[30] Ichikawa S, Motosugi U, Sato K, Shimizu T, Wakayama T, Onishi H (2021) Transient respiratory-motion artifact and scan timing during the arterial phase of gadoxetate disodium-enhanced MR imaging: the benefit of shortened acquisition and multiple arterial phase acquisition. Magn Reson Med Sci 20:280–289. 10.2463/mrms.mp.2020-0064 32863326 10.2463/mrms.mp.2020-0064 PMC8424022

