# Supplementary material for: AI-augmented reconstruction provides improved image quality and enables shorter breath-holds in contrast-enhanced liver MRI
Source: Eur Radiol Exp. 2025 May 1;9:46. doi: 10.1186/s41747-025-00582-1 (PMC12045906; doi:10.1186/s41747-025-00582-1)
Supplement: Supplementary file 1 — Additional file 1: Supplementary Table 1. Image quality scores for two readers. Supplementary Table 2. Number of lesions detected per patient, for 12-s NN+ID compared with 17-s acquisition standard reconstruction for two readers. [file 41747_2025_582_MOESM1_ESM.pdf]

AI-augmented reconstruction provides improved image quality and enables shorter breath-holds in contrast-enhanced liver MRI

ELECTRONIC SUPPLEMENTARY MATERIAL

	Reader 1			Reader 2		
	17-s NN+ID	12-s NN+ID	17-s standard	17-s NN+ID	12-s NN+ID	17-s standard
Overall image quality	4 (3 to 4)	3 (3 to 4)	2 (2 to 3)	4 (3 to 4)	4 (3 to 4)	2 (2 to 3)
Contrast-to-noise ratio (CNR)	3 (3 to 4)	3 (3 to 4)	2 (2 to 3)	4 (3 to 4)	4 (3 to 4)	2 (1 to 2)
Lesion edge sharpness	3 (3 to 4)	3 (3 to 4)	2 (2 to 3)	4 (3 to 4)	4 (3 to 4)	2 (1 to 3)
Vessel edge sharpness	4 (3 to 4)	4 (3 to 4)	2 (2 to 3)	4 (3 to 4)	4 (3 to 4)	2 (2 to 3)
Respiratory motion artefacts	3 (3 to 3)	3 (3 to 3)	3 (2 to 3)	4 (3 to 4)	4 (3 to 4)	3 (3 to 4)

Supplementary Table S1. Image quality scores for two readers.
Results show median scores (interquartile range). Data as shown in Fig. 4. *ID* Iterative denoising, *NN* Neural networks.

Supplementary Table S2. Number of lesions detected per patient, for 12-s NN+ID compared with 17-s acquisition standard reconstruction for two readers.

		12-s NN+ID	17-s standard	*p*-value	Number of patients where number of lesions differed between 12-s NN+ID and 17-s standard series (median difference in number of lesions recorded [IQR])
Reader 1	Number of lesions recorded per patient, median (IQR)	2 (1 to 5)	2 (1 to 4)	0.590	34/45 0 (-1 to 1)
	Total number of lesions recorded (total from 45 patients)	183	214	n/a	
	Diameter of smallest lesion recorded / mm (median, IQR)	4.7 (2.7 to 8.1)	4.3 (3.2 to 6.0)	0.920	
Reader 2	Number of lesions recorded per patient (median, IQR)	2 (1 to 5)	2 (1 to 5)	0.202	24/45 0 (-1 to 0)
	Total number of lesions recorded (total from 45 patients)	208	195	n/a	
	Diameter of smallest lesion recorded / mm (median, IQR)	3.7 (2.2 to 7.5)	4.5 (2.8 to 10.0)	0.024	

Results show median number of lesions (interquartile range, IQR). Of 50 patients, 50 were included in analysis of lesion detection. The *p*-values show results from Wilcoxon signed rank test for number of lesions and diameter of smallest lesion recorded using 12-s NN+ID compared with 17-s acquisition standard reconstruction, with Bonferroni corrected threshold for significance 0.05/4=0.013. The difference in number of lesions recorded is shown as the number in the 17-s standard series minus the number in the 12-s NN+ID series (*i.e.*, negative numbers) denotes more lesions recorded in 12-s NN+ID series and *vice versa*. *ID* = Iterative denoising, *NN* = Neural networks
